# Counselling for prenatal anomaly screening to migrant women in the Netherlands: An interview study of primary care midwives’ perceived barriers with client–midwife communication

**DOI:** 10.18332/ejm/147911

**Published:** 2022-05-19

**Authors:** Isabel Koopmanschap, Linda Martin, Janneke T. Gitsels - van der Wal, Jeanine Suurmond

**Affiliations:** 1 Amsterdam University Medical Center, Department of Public and Occupational Health, University of Amsterdam, Amsterdam, Netherlands; 2 Department of Midwifery Science, Academy Midwifery Amsterdam and Groningen (AVAG), Amsterdam Public Health Research Institute, Amsterdam University Medical Center, Vrije Universiteit Amsterdam, Amsterdam, Netherlands

**Keywords:** genetic counselling, prenatal diagnosis, midwives’ experiences, migrant pregnant women, client-midwife communication, ethnic inequalities

## Abstract

**INTRODUCTION:**

Large ethnic inequalities exist in the prenatal screening offer, counselling, informed decision-making, and uptake of prenatal anomaly tests. More insight into midwives’ experiences with offering prenatal counselling to migrant women may provide better insight into the origins and consequences of these ethnic inequalities.

**METHODS:**

We conducted interviews with 12 midwives certified as counsellors for prenatal anomaly screening for women they identified as migrants. Interviews were analyzed using thematic analysis.

**RESULTS:**

Midwives reported most difficulties in communicating with women of ‘non-western migrant background’, which include first- and second-generation migrants from Africa, Latin-America, Asia, and Turkey. They experienced barriers in communication related to linguistics, health literacy, sociocultural and religious differences, with midwife stereotyping affecting all three aspects of counselling: health education, decision-making support, and the client–midwife relation. Health education was difficult because of language barriers and low health-literacy of clients, decision-making support was hampered by sociocultural and religious midwife–client differences, and client–midwife relations were under pressure due to sociocultural and religious midwife–client differences and midwife stereotyping.

**CONCLUSIONS:**

Barriers to optimal communication seem to contribute to suboptimal counselling, especially for women of ‘non-western migrant background’. Client–midwife communication thus potentially adds to the ethnic disparities observed in the offer of and informed decision-making about prenatal anomaly screening in the Netherlands. The quality of prenatal counselling for women from all ethnic backgrounds might be improved by addressing linguistic, health literacy, sociocultural and religious barriers in future training and continuing education of prenatal counsellors.

## INTRODUCTION

In 2007, a national prenatal anomaly screening program was introduced in standard prenatal care in the Netherlands^1^. The legislative framework supporting the program dictates that all pregnant women in the Netherlands must have equal access to it. Nevertheless, large ethnic inequalities have been observed in information provision, counselling, informed decision-making and uptake of prenatal screening in the Netherlands^2-6^.

In the Netherlands, 25% of the population today consists of individuals of non-Dutch origin^7^. Someone is considered of non-Dutch origin if he/she, or at least one parent, was born abroad. More than half of the residents of this group is considered to be of ‘non-western’ origin, which include people of African, South American and Asian (including Turkey, but excluding Indonesia and Japan) descent^8^. The largest groups of non-western migrants living in the Netherlands are Turks (414181 people, 47.3% first and 52.7% second generation), Moroccans (406582 people, 42.1% first and 57.9% second generation) and Surinamese (355559 people, 49.7% first and 50.3% second generation)^9^. First generation Moroccan and Turkish migrants moved to the Netherlands as workers in the 1960s and 1970s. Between 1975 and 1980 the first generations of Surinamese people migrated to the Netherlands, after Suriname obtained its independence. The four largest groups of refugees with a non-western migrant background in the Netherlands are Iraqis, Afghans, Syrians, and Somalis^8^. Someone is considered of ‘western’ descent if he/she, or at least one parent, was born in Europe, North America, Indonesia, Japan or Oceania^7^.

Studies analyzing ethnic inequalities in prenatal screening in the Netherlands focus primarily on the four largest ‘non-western’ ethnic groups. A register-based study among 30095 clients, for example, demonstrated that Surinamese, Turkish, Moroccan, Cape Verdean and Antillean women are less likely to be offered information and counselling about prenatal anomaly screening than ethnic Dutch women, especially when they are first-generation migrants and/or have low levels of Dutch language proficiency^6^. Additionally, some evidence indicates that women with a Turkish and Surinamese migrant background less often understand the prenatal screening information compared to ethnic Dutch women^5^ and less often make an informed decision^4^. Moreover, large ethnic disparities in uptake of prenatal screening were found between Moroccan, Turkish, Antillean and Surinamese women on the one hand and ethnic Dutch women on the other hand, even after adjustment for differences in socioeconomic background and age^2,3^.

The Dutch prenatal screening program aims to enable pregnant women and their partners to make an autonomous informed decision about whether to participate in prenatal screening for congenital anomalies^10^. To facilitate this, counselling for prenatal anomaly screening comprises three components: health education (HE), which entails information provision about target anomalies, test characteristics and test procedures, and decision-making support (DMS), which involves discussion and reflection on clients’ values. Both functions of counselling are facilitated by the third component: a good client–midwife relation (CMR)^11^. In most cases (85%), trained primary care midwives provide counselling on prenatal anomaly screening in the Netherlands^12^. The screening offer consists of first-trimester screening for Down, Edwards and Patau syndromes through the use of the non-invasive prenatal test (NIPT) or the first trimester combined test (FCT), and second-trimester screening for structural anomalies through a fetal anomaly scan (FAS). The NIPT is offered alongside FCT, but as the NIPT has proved much more accurate, women rarely opt for FCT nowadays^13^. Therefore, this study only discusses NIPT and FAS from this point onwards.

Prior studies have proposed various explanations for the existence of ethnic inequalities in prenatal screening in the Netherlands as well as in other countries. Some studies suggest that differences in informed decision-making and uptake reflect attitudes of migrant women towards testing, such as lack of interest in prenatal screening^5^, low risk-perception^3^, and religious identity^3^. A Dutch study^3^, for example, suggests that women of Surinamese and Turkish backgrounds are less likely to participate in prenatal screening due to accepting ‘what God gives’, and low perceived age-related risk of having a child with Down syndrome. However, other studies from the Netherlands and the UK indicate that lower uptake of prenatal screening in migrant women, rather than reflecting attitudes towards prenatal testing, are related to inequalities in the offer of information and counselling as well as linguistic and education barriers in access to prenatal screening information materials^6,14-17^. Moreover, they indicate that differences in the offer of information and counselling are related to delays in being able to access prenatal care by migrant women^6,13^, especially for those with low levels of Dutch language proficiency, and low education level^18^. Finally, recent findings suggest that the obliged co-payment for the NIPT of 175 euro raises differences in test uptake between women living in socioeconomically disadvantaged neighborhoods (20.3%) and those living in more advantaged neighborhoods (47.6%)^19^. Migrants of all non-western migration backgrounds generally have a lower household income than the average income in the Netherlands^20^. The costs may therefore more often raise a barrier to opt for NIPT for migrant women of ‘non-western background’ than ethnic Dutch women and migrants of ‘western background’.

Although provider-related communication barriers such as ethnic stereotyping by midwives and lack of intercultural competences have been hypothesized as possible explanations for ethnic inequalities^6,14,16^, and to our knowledge they have not yet been investigated. Consequently, barriers concerning communication during prenatal counselling and their impact on the three counselling functions remain to be elucidated. To fill this gap, the present study aimed to investigate primary care midwives’ perceptions of client–midwife communication during counselling for prenatal anomaly screening with migrant women.

## METHODS

### Study design and population

This study used semi-structured interviews among primary care midwives in the Netherlands. Data collection took place between March and May 2019. Midwives were eligible for participation if they were certified as counsellors, if counselling for prenatal anomaly screening was part of their usual work (at least 50 counselling sessions a year)^21^, and if their clientele consisted of a considerable number of migrant women.

### Data collection

We recruited participants using purposive sampling, focusing on midwifery practices representing diverse geographical locations in the Netherlands. Previous studies have shown large differences in prenatal screening uptake of NIPT between four regions (North, West, South, East) of the Netherlands^22^. We, therefore, targeted potential participating practices in all four regions. First, we identified cities with relatively high proportions of residents with a migrant background (cut-off point 15%)^9^. For the Northern and the Southern provinces, where cities are inhabited by generally lower percentages of migrants, we used a cut-off point of 10% to ensure inclusion. Second, within those cities, we identified neighborhoods with high percentages of migrants (cut-off point 20%), using national registers of residents at the neighborhood level^23^. In every city, we picked the neighborhood with the highest percentage of migrants and checked for midwifery practices located within or near that neighborhood. We recruited midwifery practices matching the criteria mentioned above through the professional national network of the department of Midwifery science of the Amsterdam University Medical Center (UMC) and by directly contacting them through email. The information letter specified that the study aimed to understand prenatal counselling of migrant women, intentionally leaving open the term ‘migrant’.

Interviews were conducted via telephone (n=11) or face-to-face in the midwifery practice (n=1), following participants’ preference. A semi-structured interview guide was developed, based on the literature on intercultural client–provider communication (Table 1). At the start of the interview, we asked midwives to estimate the proportion of migrant women they counselled. Moreover, we asked them to specify when they talked about a ‘migrant woman’. Throughout the interviews, we asked participants to specify what women they referred to (e.g. migrant generation, socioeconomic background, and ethnic background) when they talked about a particular topic or situation. Interviews lasted approximately 1 hour, ranging in length from 41 minutes to 140 minutes, were audio-recorded and transcribed. Transcripts were labelled with unique random identification numbers. Participants, practices, and places were assigned random aliases. All participants received oral and written information about the purpose of the study before the interview. They were informed about confidentiality and the possibility of withdrawing at any time without giving a reason. They gave their oral and written informed consent for participation. Following Dutch law, the medical ethics committee of the Amsterdam UMC deemed the study exempt from ethical review.

**Table 1:** Topics list used for the interviews

*Topics*
***1.*** What are your experiences with counselling women with a migrant background compared to native Dutch women?
***2.*** Do you ever experience barriers in providing prenatal counselling to women with a migrant background? If so, what barriers do you experience?
***3.*** What are your experiences with the impact of a language barrier (if any) on the communication during the counselling session?
***4.*** How does the religious identity of the client influence the communication during the counselling session?
***5.*** To what extent do you take account of the sociocultural background of your client when counselling?
***6.*** What are the effects of the current prenatal screening regulations on the way you provide counselling to women with a migrant background?

### Data analysis

The transcripts were coded and analyzed using thematic analysis^24^. First, five transcripts were read and re-read, and divided into fragments. Fragments were compared, grouped into themes, and labelled with preliminary codes. First, IK and JS discussed the themes and preliminary codes until they reached consensus. Second, IK read all transcripts and tried to refine the themes and discover relationships between them. This involved retrieving and comparing fragments assigned to particular codes, finding negative evidence, combining and refining themes and organizing them into main themes and subthemes. Third, the themes and subthemes were refined, core concepts were identified and abstracted, and relations between core concepts were established. To reach inter-subjectivity of the results, two researchers (IK and JS) independently coded the first transcript.

## RESULTS

### Participant characteristics

Table 2 provides sociodemographic characteristics of the participating midwives. Twelve female midwives from eight midwifery practices across The Netherlands participated. Respondents ranged in age from 27 to 60 years, with an average age of 43 years. Most respondents were categorized as ethnic Dutch (n=10). The thematic analysis elicited four major themes: 1) language barrier, 2) low health-literacy, 3) sociocultural and religious differences between midwife and client, and 4) midwife stereotyping.

**Table 2:** Characteristics of midwives in the interview study

*Characteristics*	*n (%)^a^*
**Geographical location**	
North	2
East	1
South	1
West	8
**Work experience** (years), mean (range)	14.9 (1–34)
≤2 years	2 (18.2)
3–11 years	3 (27.3)
≥12 years	6 (54.5)
**Counselling experience** (years)^b^, mean ± SD (range)	7.3 ± 5.1 (1–12)
≤2 years	3
3–11 years	2
≥12 years	6
**Age** (years), mean ± SD (range)	43.3 ± 11.7 (27–60)
**Ethnicity**	
Dutch	10 (83.4)
Other western	1 (8.3)
Non-western	1 (8.3)
**Religious background**	
None	7 (58.3)
Christian	3 (25.0)
Muslim	2 (16.6)
Other	–

a Due to missing and inapplicable answers the n can vary from variable to variable. Valid percentages are shown.

b Counseling experience was measured from 2007, when the Dutch prenatal screening program was implemented and when midwives became obliged to provide counseling to all pregnant women in the Netherlands. Nevertheless, midwives have provided information about prenatal screening and diagnosis already for many years before 2007 on the Triple test, amniocentesis and CVS to women with a high risk of congenital anomalies and low risk women who explicitly asked for information. However, this information provision did not involve counseling.

When we asked midwives to define a ‘migrant woman’, all acknowledged the broad diversity of women this term entailed in terms of ethnicity, language proficiency, and socioeconomic status. However, when talking about the barriers encountered during counselling, midwives often narrowed down the term ‘migrant woman’ to denote women with ‘a non-western migrant background’.

### Language barrier

The difficulty midwives mentioned most often concerned communicating with clients with a migrant background and not proficient in Dutch or English. A language barrier limited the ability to exchange the information necessary for the client to fully understand the test offer, particularly affecting HE. Midwives often simplified the information, which could lead to insensitive information provision. Especially when it concerned the topic of pregnancy termination, some midwives considered it difficult to bring across information without scaring off their clients and feared that clients would misinterpret the aim of the screening tests:

*‘[I]t's a very delicate topic to explain if you are not fully proficient in the language [of your client]… It is perfectly possible that they misunderstand the information and think, ‘If you take a test, you need to terminate your pregnancy.’* (MW01)

In case of a language barrier, most midwives were also concerned that the information they provided was oversimplified, especially in the case of NIPT. Some test characteristics, e.g. it is a blood test, sequencing both fetal and maternal DNA and it is a risk assessment, were particularly difficult to explain to clients not proficient in Dutch. These details were easily omitted from counselling to make the test easier to explain. As such, counsellors felt they were unable to provide sufficient information to enable an autonomous and well-informed choice:

*‘[In case of a language barrier] you just do not explain everything about DNA, the placenta, sensitivity, and specificity. You rather say something like, “the NIPT is more accurate”. And then they may ask “Better?”, “Yes, better”, although you actually think, “I am not supposed to say that”.’* (MW03)

In contrast, no difficulties were experienced when explaining the FAS. As an ultrasound is more tangible than a genetic test, midwives found this test much easier to explain to women not proficient in Dutch:

*‘People [with low Dutch language proficiency] know that with an ultrasound you can see something, for example spina bifida or heart defects. So, it is much easier to explain.’* (MW05)

To overcome a language barrier, counsellors often used informal interpreters. Yet this also brought along additional problems, such as the risk of biased translations. Despite the issues experienced with informal interpreters, professional interpreter services were rarely used. Most midwives mentioned that they were too expensive. In the Netherlands, midwifery practices are obliged to pay for telephone interpreters themselves since 2012^25^. For a consultation of 30 minutes, the charges are 71 euros for telephone interpretation and 92 euros for face-to-face interpretation, excluding tax^26^. Although these costs are not directly reimbursed by the government, midwifery practices receive an extra reimbursement of 346 euros for clients living in disadvantaged areas, which is intended to cover extra expenses made during the entire course of the pregnancy^27^.

### Low health-literacy

All midwives encountered health literacy issues among women with a non-western migrant background, particularly first generations with low education level. They indicated that the latter often lacked necessary basic knowledge about genetics, Down syndrome, and the human body. This required midwives to spend time on explaining the basic features of the tests (HE), which they were not always able to do properly within the designated time. Especially during counselling on the NIPT, due to complexity of the information, they experienced difficulties in explaining all test details:

*‘[I] sometimes notice that knowledge levels about the body are lower [among migrant women]. Then, I must explain what a placenta is or that you have DNA in your blood. Yeah, well, I can start explaining all that, but sometimes people just do not have enough background knowledge [to be able to explain a test]. That completely changes the counselling.’* (MW03)

Another challenge was when midwives asked their clients to read the information leaflets in preparation for counselling. Although these leaflets are translated in the five most spoken foreign languages (e.g. Arabic and Turkish), midwives were under the impression that the great majority of migrant women from non-western countries did not read them. Most of them referred to both first- and second-generation women, with low levels of Dutch proficiency, and low literacy levels. Some midwives found this profoundly irritating as this obliged them to spend more time on HE compared to when women came prepared. Consequently, midwives noted, counselling remained ‘superficial’ and midwives had insufficient time to discuss a client’s moral considerations towards testing and the resulting decisions (DMS):

*‘If I ask, “Have you read the booklets?” they may say “yes”, but if I then try to have an in-depth discussion, by asking questions like “Have you talked about it together? How much would you want to know about the health of your child?” I generally receive a very superficial answer that shows they actually have not read it. That makes it extremely difficult to have a real counselling session – to have an in-depth discussion.’* (MW06)

Counsellors provided several explanations for the observed lack of preparation by migrant clients. Most often they mentioned that the information was not translated into less common languages of clients, that the information was too technical, and clients’ insufficient levels of reading ability. Additionally, some midwives perceived a lack of interest as the cause of a lack of preparation. They observed that most women, independent of language proficiency or literacy levels, decided to decline NIPT before they even received information or counselling about the test. This lack of interest was not observed for FAS.

### Sociocultural and religious differences between midwife and client

Even if clients were proficient in Dutch or English and had high levels of health literacy, midwives encountered some sociocultural and religious differences between themselves and their clients, which they perceived affected decision-making. These differences did not involve women’s ethnic background per se, but rather their assumed religious identity and the assumed role of other family members in the decision-making.

When bringing up the topic of prenatal screening for the first time, all midwives noted that women who they identified as Muslim generally declined information about NIPT. This situation often resulted in an internal struggle for midwives. A few midwives indeed emphasized the importance of the so-called ‘right not to know’ and stopped giving information about NIPT immediately. Most midwives, however, also wanted their clients to know more about the test they declined, to be able to make an informed decision. Consequently, they often gave more information about NIPT than their clients asked for, which they considered as conflicting with their clients’ ‘right not to know’:

*‘If women already say upfront “I don't want that”, I will try to understand the reason why. Because I sometimes suspect that women do not have sufficient information about what they can do with it [NIPT]… And then I try to explain what the possibilities are. So, I sometimes explain more than they actually want to know.’* (MW02)

While most midwives considered religion as a valid reason for declining prenatal screening, for some a choice based on religious reasons conflicted with their vision of good counselling. They were trained to support pregnant women in making an individual and autonomous decision:

*‘I would like them [Muslim women] to make a choice of their own, instead of letting their choice depend on religion or family. I would like them to look at their own lives, as a couple, as partners. “What do we want our future to look like?”, “What kind of future do we wish for our child?”, “Can we take care of an affected child?”. That is what I would like to discuss with them.’* (MW07)

Additionally, some midwives found it difficult to deal with the influence of the client’s family in decision-making, which they often attributed to the assumed cultural background of the client. On the one hand, they wanted their clients to think about their decision on prenatal screening autonomously, without considering opinions of the family. At the same time, however, they wanted to accept their clients as they were and wanted to respect ‘their way of living’. Encouraging the client to make an autonomous decision, they feared, could unintentionally come across as discrimination or as prescribing a particular choice, potentially affecting the CMR and DMS function of counselling:

*‘You don't want people to think that you're judging them, and you don't want to give them the feeling that you're pushing them to take a test only because their family says they shouldn't opt for one. No, I want them to make a decision that they're comfortable with. But that is the difficult thing here. What if you sense that they are considering accepting testing, but they decline because their family says they should not accept? You do not want to push them so far that they think that I want them to take a test.’* (MW01)

### Midwife stereotyping

The observed use of stereotyping during counselling differed between midwives and differentially impacted on client–midwife communication. Some midwives observed that, even though they had expectations about the prenatal screening decision of a client with a non-western migrant background, these expectations would not affect the counselling. Other midwives, however, indicated purposively making use of stereotyping to attune counselling to their clients’ needs. Through experience, they had learnt to be sensitive to particular client characteristics, such as low literacy, social pressure and poor financial situation. Such characteristics could help to counsel purposively, i.e. adjust language use to the level of understanding of the client, and asking questions deemed relevant for the client in question:

*‘We know this woman is Muslim … Then I ask right away: “If your baby has one of these syndromes, do you want to take care of it, or do you want to end your pregnancy?”. I normally ask that question at the end, but I know this question is useful to ask in the beginning [in case of a Muslim client]. She will probably answer, “I won't terminate my pregnancy, because I will accept every child” … Then I do not have to explain every single detail about the NIPT when she does not want it anyway.’* (MW04)

Whilst stereotypes were mostly considered to facilitate counselling, some midwives indicated that they could also lead to bias. Especially when counselling women from particular ethnic or religious backgrounds of which most women declined prenatal testing, midwives could experience difficulties with keeping an open mind:

*‘I often counsel Eritrean women, but they never opt for prenatal screening. So, when an Eritrean lady comes in, I easily think, “Well, we're going to do the counselling, but there is a big chance that she doesn't want it anyway”… And if they decline testing, you might be more inclined to accept it right away and think, “Well, that's what I thought already”.’* (MW01)

## DISCUSSION

From the analysis, four themes affecting the client–midwife communication with clients with a migrant background emerged: language barrier, low health-literacy, sociocultural and religious differences between client and midwife, and midwife stereotyping. The perceived barriers affected all three aspects of prenatal counselling – HE, DMS and CMR – thereby potentially limiting the possibility of women with a non-western migrant background to make an informed choice. Midwives indicated that they primarily perceived barriers with regard to women of ‘non-western’ origin.

In support of prior research conducted in midwifery care^28-30^, this study found that language difficulties are the most prominent and most common issue raised by counsellors when counselling women with a non-western migrant background on the NIPT. Language difficulties in client–midwife communication were perceived to lead to incomplete, insensitive, and oversimplified information exchange, which generally resulted in poorer HE. Language barriers may thus partly explain previously observed ethnic differences in women’s levels of understanding of prenatal screening information and informed decision-making in the Netherlands^2^.

The findings also suggest that, even in the absence of a language barrier, other communication factors hinder optimal HE on the NIPT to women with a non-western migrant background. Midwives reported ‘low health-literacy’, in terms of a lack of background knowledge and preparation by women, as negatively affecting counselling, which they partly ascribed to a lack of interest in prenatal screening on the part of their clients. A seemingly lack of preparation and interest, however, can also originate from an inappropriate offer of information^31^. In response to an observed lack of preparation, midwives prioritized HE, thereby leaving little time for DMS, which is in fact deemed the most crucial part of prenatal counselling^32,33^. Providing too much and too complex information works counterproductively and may obscure the information needs of clients with low health-literacy levels^34,35^. Instead, prioritizing a discussion of clients’ values and preferences regarding pregnancy termination may be more relevant than providing all technical details about the tests^32^. Moreover, differentiating in information exchange modes, e.g. reading the information leaflet together rather than providing standard counselling, can help to tailor counselling to the individual information needs and learning styles of different clients.

Corroborating earlier research, the finding that midwives perceived barriers to communication related to ‘sociocultural and religious differences between themselves and their clients suggests that counsellors consider it challenging and uncomfortable to relate to clients’ values and beliefs in counselling^32^ and sometimes presume that their clients are acting out of undue pressure when the family has a role in the decision-making^35^. These challenges may result in struggles for midwives to remain value-free and non-directive towards clients’ decision-making^36^. These struggles indicate that midwives may rely on a view of the client as an autonomous decision-maker who makes decisions independent of her social environment. There are, however, cultural differences in attitudes toward patient autonomy and some ethnic groups are more likely to embrace a family-centered model of decision-making^37^. Counsellors may be insufficiently aware of the existence of these different decision-making models and may be unaware that women who hold a secular world view are equally directed by their values and beliefs when making a prenatal screening decision.

This is the first study showing that midwives experience barriers in counselling for prenatal screening related to their own ‘stereotyping’. One example of stereotyping behavior was that women of some ethnic and religious backgrounds were thought to be more inclined to decline screening information and counselling. Stereotyping behaviors, either intended or unintended^38^, have detrimental effects on the quality of prenatal counselling, and midwives’ ability to tailor care to individual needs of women. Prior evidence indicates that stereotypes and prejudiced behavior experienced by women can discourage them from engaging in consultations^29^, and can lead to insufficient information provision about treatment options^38^. A Swedish interview study, for example, found that Somali-born parents were stereotyped as ‘embracing non-medicalized approaches to pregnancy’, and therefore did not always receive information about topics such as pain relief^38^. Our results describe a similar potential implication for the offer of information and counselling to women. When midwives assume that women with particular ethnic backgrounds and religious identities might be less interested in prenatal screening, which was described by some of the midwives, this may lead to inequalities in the offer of prenatal screening and consequently in unequal opportunities for making an informed choice. Other possible explanations for women’s hesitancy to receive information or counselling, including low health-literacy, may be overlooked.

In contrast to NIPT, this study found that midwives encountered fewer barriers in providing counselling about FAS to women of non-western migrant backgrounds, even those with low language proficiency and low levels of health literacy. This observed difference between the two tests suggests that counsellors approach the NIPT and FAS as separate entities during prenatal counselling. The Dutch guidelines on prenatal screening, however, prescribe a generic offer of prenatal screening. This means that prenatal counselling should highlight the aim of prenatal screening, i.e. finding out more about congenital anomalies in the unborn child rather than focusing on the specific tests to reach this aim.

### Implications for practice

The difficulties that midwives face in counselling migrant women with low Dutch language proficiency and low health-literacy levels seem to suggest that counsellors need more training tailored to these specific challenges. Such training should enable midwives in making complex prenatal screening information accessible to women with various levels of background knowledge, literacy levels and Dutch language proficiency. For this purpose, existing training materials and prenatal screening guidelines^20^ need to better support midwives in deciding when and how to use what type of information material, in order to align with the needs and learning style of each individual client.

The barriers related to ‘sociocultural and religious differences’ and ‘stereotyping’ we found in this study also indicate that midwives struggle with providing person-centered counselling when women fail to meet their normative ideals of autonomous and informed decision-making. Person-centered counselling has been suggested as an approach to counteract stereotyping and for providing help to sensitize providers to their own behavior and unintentional prejudices^38^. Person-centered care includes self-reflection of midwives regarding how they value their own existential life questions and how these may influence encounters with clients^32^. Continuous education may provide a good opportunity to reflect on issues such as the meaning of ‘autonomy’ in decision-making. Using a relational conceptualization of autonomy^39^ can provide midwives with tools for appreciating and dealing with a wide range of decision-making strategies of clients, including strategies that involve family members.

Future studies should include migrant women’s perspectives and expectations to gain a more complete picture of ethnic inequalities in client–midwife communication. Although previous work has focused on women’s views on religion in prenatal counselling^40,41^, their experiences with ethnic discrimination and culturally competent communication remain understudied. Additionally, observational research of counselling sessions is necessary to provide better insight into ethnic biases by midwives.

### Limitations

A limitation of our study is a potential selection bias. The interviewed midwives may have had an interest in the study and therefore may have been relatively good in counselling clients with a migrant background. Midwives who may be less favorable towards clients with a migrant background may experience other or more intense barriers. Moreover, the majority of participating midwives were Dutch, therefore, not reflecting the experiences of midwives from other ethnic backgrounds. Previous studies in other healthcare settings show that ethnic concordant consultations score generally better on patient–provider communication outcomes and patient satisfaction than ethnic-discordant interactions, even when controlling for socioeconomic status^42^. Hence, ethnic minority midwives may experience fewer or different communication barriers when counselling women from similar ethnic and linguistic backgrounds. A final limitation of our study is that we followed midwives’ own definition of ‘migrant women’ during the interviews. Previous studies find, for example, that physicians tend to ascribe ‘difficulties’ they experience with migrant patients to their patients’ assumed cultural background, while they describe them to personal circumstances and characteristics in patients of the ethnic majority group^43^. Thus, our study may present an overestimation of communication barriers in ‘non-western’ ethnic minority groups, while potentially leading to underestimation in ethnic Dutch and European migrant groups.

## CONCLUSIONS

Our study suggests that client–midwife communication plays a role in ethnic inequalities in prenatal counselling in the Netherlands. Midwives experience difficulties in providing prenatal counselling for prenatal anomaly screening to women with a non-western migrant background due to linguistic, health literacy and sociocultural and religious barriers. These barriers negatively affect HE, DMS and CMR, potentially limiting the possibility of women with a non-western migrant background to make an informed choice.

## Data Availability

The data supporting this research cannot be made available for privacy reasons.
